# Identification of the target DNA sequence and characterization of DNA binding features of HlyU, and suggestion of a redox switch for *hlyA* expression in the human pathogen *Vibrio cholerae* from *in silico* studies

**DOI:** 10.1093/nar/gku1319

**Published:** 2015-01-20

**Authors:** Debadrita Mukherjee, Aritrika Pal, Devlina Chakravarty, Pinak Chakrabarti

**Affiliations:** 1 Bioinformatics Centre, Bose Institute, P1/12CIT Scheme VIIM, Kolkata 700054, India; 2 Department of Biochemistry, Bose Institute, P1/12 CIT Scheme VIIM, Kolkata 700054, India

## Abstract

HlyU, a transcriptional regulator common in many *Vibrio* species, activates the hemolysin gene *hlyA* in *Vibrio cholerae*, the *rtxA1* operon in *Vibrio vulnificus* and the genes of *plp*-*vah1* and *rtxACHBDE* gene clusters in *Vibrio anguillarum*. The protein is also proposed to be a potential global virulence regulator for *V. cholerae* and *V. vulnificus*. Mechanisms of gene control by HlyU in *V. vulnificus* and *V. anguillarum* are reported. However, detailed elucidation of the interaction of HlyU in *V. cholerae* with its target DNA at the molecular level is not available. Here we report a 17-bp imperfect palindrome sequence, 5′-TAATTCAGACTAAATTA-3′, 173 bp upstream of *hlyA* promoter, as the binding site of HlyU. This winged helix-turn-helix protein binds necessarily as a dimer with the recognition helices contacting the major grooves and the β-sheet wings, the minor grooves. Such interactions enhance *hlyA* promoter activity *in vivo*. Mutations affecting dimerization as well as those in the DNA–protein interface hamper DNA binding and transcription regulation. Molecular dynamic simulations show hydrogen bonding patterns involving residues at the mutation sites and confirmed their importance in DNA binding. On binding to HlyU, DNA deviates by ∼68º from linearity. Dynamics also suggest a possible redox control in HlyU.

## INTRODUCTION

The pathogenesis of cholera, caused by *Vibrio cholerae*, is a complex process involving a number of factors accounting for the establishment of the pathogen in the epithelium of the small intestine and production of the enterotoxin that disrupts ion transport by intestinal epithelial cells. The major features of the pathogenesis of cholera are well established, with toxin coregulated pilus (TCP), cholera toxin (CTX), accessory cholera enterotoxin (ACE), zona occludens toxin (ZOT) and hemolysin (HlyA) having been identified as the key virulence factors. HlyA, a cytotoxin as well as an enterotoxin (1–4), has been implicated as a virulence determinant in the infant mouse cholera model (4). Numerous investigations proved that the hemolytic (Hly+) *V. cholerae* strains were of the El Tor biotype, whereas classical biotype strains were always found to be non-hemolytic (Hly−). In the recent past, the proportion of hemolytic *V. cholerae* isolates in Africa has increased dramatically (5), and the series of outbreaks of cholera along the U.S. Gulf Coast beginning in 1973 was due to a strongly hemolytic El Tor strain of *V. cholerae* (6,7). One of the possible pathogenicity factors of the *V. cholerae* strains that are not able to produce CTX but still cause gastrointestinal disorders (8,9) has been implicated to be the El Tor HlyA (1,10).

Besides the much discussed virulence regulatory systems, like ToxR, Fur, etc., in *V. cholerae*, the HlyU regulatory system has been found to be involved in *hlyA* gene expression and probably expression of other virulence factors that are required for the efficient colonization of the host small intestine by this organism (11,12). A *hlyU* mutant has been found to be deficient in the production of HlyA and Hcp (a 28 kDa secreted HlyA co-regulated protein, a part of the Type 6 secretion system) (13), as well as defective in colonization, with a 100-fold increase in the LD_50_ in the infant mouse cholera model (12). HlyU of *V. cholerae*, (HlyU_Vc) is known to belong to the SmtB/ArsR family of regulatory proteins including NolR of *Rhizobium meliloti*, SmtB of *Synechococcus sp*., ArsR of *Staphylococcus aureus* (14). The homology model of the protein and the very recently solved crystal structure (Protein Data Bank (PDB) ID: 4OOI, (15)) show that the protein is a homodimer with a winged helix-turn-helix (wHTH) motif (Supplementary Figure S1). Unlike the other metallorepressor members of its family, HlyU_Vc is a transcription activator (16) lacking any metal binding domain (15).

Though Williams and Manning demonstrated that a 710-bp fragment spanning the predicted promoter in the 5′ region of *hlyA* in conjunction with HlyU_Vc increased chloramphenicol acetyl transferase activity and HlyA production in *Escherichia coli* (16), no further findings regarding the DNA-HlyU_Vc interactions have been documented in the literature to date. Here we report studies pertaining to direct biochemical evidence of HlyU_Vc binding to the region upstream of *hlyA* promoter region using gel shift assays and *in vivo* reporter gene assays. We have revealed the nucleotide bases interacting with HlyU_Vc to specificity using DNase I footprinting studies. We have also endeavored to dissect the protein structure–function relation through mutational approaches and identify the actual residues of HlyU_Vc that are essential for dimerization and DNA-binding activity. The involvement of both the major and the minor grooves of the double helix and the probable conformational changes of the nucleic acid in the DNA-HlyU_Vc complex have also been reported. Additionally, we have carried out molecular dynamic (MD) simulations on a DNA-HlyU_Vc model. The simulations validate the solution studies as well as suggest a possible redox role of the Cys residues in DNA binding by HlyU_Vc.

## MATERIALS AND METHODS

### Bacterial strains, plasmids, radioisotopes and other chemicals

*V. cholerae* El Tor strain N16961 was a gift from Dr R. Nandy (National Institute of Cholera and Enteric Diseases, Kolkata). The specialized cloning vector pBend4 was provided by Dr S. Dasgupta (Bose Institute, Kolkata). All the bacterial strains, plasmids and clones used in the study are provided in Supplementary Table S1. [γ^32^P]-ATP was obtained from Board of Radiation and Isotope Technology, India (1 milli Curie ml^−1^). X-ray films and film developing chemicals were purchased from Kodak. *ortho*-Nitrophenol galactoside (ONPG) is from Calbiochem. All enzymes are from Thermo Scientific. All other chemicals were from Merck and of molecular biology grade.

### Designing of DNA fragments for electrophoretic mobility shift assay

About −582 to +246-bp DNA stretch spanning the *hlyA* transcription start site was considered for DNA binding studies. This region was initially divided into four overlapping DNA fragments (Table 1), and the corresponding forward and reverse primer sequences were designed according to the published genomic DNA sequence of *V. cholerae* N16961. Later, other fragments, DNA 5 and DNA 6 (Table 1), were generated as stated above.

**Table 1.** DNA fragments used for binding studies

Fragment Name	DNA region with respect to the transcriptional start site (length in parentheses)
DNA 1	−331 to −582 bp (252 bp)
DNA 2	−122 to −372 bp (251 bp)
DNA 3	−185 to +48 bp (233 bp)
DNA 4	−8 to +246 (254 bp)
DNA 5	−127 to −1 (127 bp)
DNA 6	−78 to −282 bp (205 bp)
DNA 7	−282 to −1 (282 bp)

### DNA manipulations and sequence analysis

Plasmid DNA isolation, gel extraction of restriction-digested DNA fragments, polymerase chain reaction (PCR)-purified fragments, etc., were done using protocols provided by the manufacturers (Qiagen; Fermentas GmbH). DNA estimation was done by reading absorbance at 260 nm using spectrophotometer (UV 1800 Shimadzu Spectrophotometer). Competent *E. coli* cell preparation and plasmid DNA transformation was done using standard calcium chloride and heat shock method. Sequencing of PCR-made DNA fragments was done by Sanger's method (3130XL Genetic Analyzer, Applied Biosciences). DNA fragments were labeled at the 5′ end with [γP^32^] ATP using polynucleotide kinase exchange reaction.

### Purification of HlyU_Vc

For over-expression of the proteins—wild-type HlyU_Vc and the mutants—competent cells of *E. coli* strain BL21 (DE3) were transformed with the corresponding constructs. One colony from each was transferred from the plate into 50 ml Luria–Bertani (LB) broth containing 50 μg ml^−1^ kanamycin for overnight growth at 37°C. Over-expression of all the proteins was achieved by growing cells in LB broth at 37°C to an OD_590_ of ∼0.5 followed by induction with 0.5 mM isopropyl-β-thiogalactopyranoside (IPTG) at 37ºC for 4 h. The cells were lysed under native conditions, and the protein was purified by using the nickel-nitrilotriacetic acid (Ni-NTA) resin (Qiagen) for affinity chromatography. The N-terminal 6X-His tag was removed using thrombin.

### Electrophoretic mobility shift assay and DNase I footprinting

DNA–protein mixes of 20 μl were prepared by adding radio-labeled probe of 1 nM with various amounts of protein in 1X binding buffer (1 mM Tris, 6 mM NaCl, 0.5 mM MgCl_2_, 0.01 mM EDTA, 0.1 mM CaCl_2_, 0.2% glycerol) and 0.03 mg ml^−1^ of Salmon sperm DNA (a non-specific DNA competitor to negate non-specific DNA–protein interactions) for 1 h at 4°C. 6% native polyacrylamide gel using Tris Borate EDTA (TBE) was used for all the electrophoretic mobility shift assays (EMSAs) and complexes electrophoresed at 75 V at 4ºC for requisite time periods. The gel was pre-equilibrated by electrophoresing at 60 V at 4ºC for 45 min.

For DNase I footprinting analysis, ∼10 nM-labeled DNA was incubated with various amounts of HlyU_Vc for 1 h at 4°C. Then, 0.15 unit of DNase I from Sigma was added and the mixture incubated for 15 min at room temperature. The digestion was stopped by adding DNase I stop solution (50 mM Tris (pH 8.0), 50 mM EDTA, 2% (wt/vol) sodium dodecyl sulphate (SDS), 0.4 mg ml^−1^ proteinase K). DNA was then precipitated using ethanol and sodium acetate and then washed with ethanol and dried. Digested DNA fragments were resuspended in loading buffer (98% vol/vol) deionized formamide, 10 mM EDTA, 0.025% wt/vol xylene cyanol and 0.025% wt/vol bromophenol blue, boiled for 5 min, chilled rapidly and separated by gel electrophoresis on 8% (wt/vol) Urea-TBE polyacrylamide sequencing gel at 1300 V for 3.5–4 h. An A+G (using formic acid and piperidine) and a G ladder (using dimethyl sulphate and piperidine) were prepared with 10 nM of labeled DNA and analyzed along with digested DNA. The gels were imaged using a phosphor imager (Typhoon trio +).

### Site-directed mutagenesis

All the desired mutations were carried out using QuikChange II Site-directed mutagenesis kit (Stratagene). The plasmid clone for *hlyU_Vc* gene in pET28a or pET23a vector (pET28a-HlyU_Vc, pET23a-HlyU_Vc; Supplementary Table S1) was used as the template for site-directed mutagenesis PCR using the corresponding primers. The wild-type templates in the PCR product mix were digested away with DpnI and the final product was transformed into *E. coli* XLIB cells. The plasmids for the mutant proteins (pET28a- XnY, pET23a- XnY; Supplementary Table S1) were extracted from the screened colonies and sequenced for the correct mutations.

### Size exclusion chromatography

Analytical gel filtration experiments were carried out in an HPLC system AKTA prime Plus. 500 μg protein was injected at a time. The column was pre-equilibrated with 50 mM sodium phosphate and 200 mM sodium chloride buffer, pH 6.8. Bovine serum albumin (66.5 kDa), RNase A (13.7 kDa), chymotrypsin (24.8 kDa) and ovalbumin (44.5 kDa) were used as molecular weight markers. The void volume was calculated by running blue Dextran. The molecular weight of the sample proteins was calculated from the standard plot of *R_f_* versus molecular weight, *M_w_*, generated from the marker proteins.

### β-galactosidase assay

*E. coli* BL21 (DE3) cells were transformed with the two plasmids: one bearing the *hlyU_Vc* gene under the control of T7 promoter (pET23a-HlyU_Vc) with Amp^R^ and the other, pDA1a containing DNA 7 (Table 1) inserted upstream of the *lacZ* gene with Kan^R^ (Supplementary Table S1). The double antibiotic resistant colonies were cultured overnight at 37°C in LB broth. Similar transformations were done with pDA1a and each of the pET23a-XnY plasmids (Supplementary Table S1) encoding L25D, L98D, Q63A and Y85A mutant proteins. A control experiment was also carried out with *E. coli* cells carrying the empty vector pET23a and pDA1a. Secondary cultures were grown for 5 h from the overnight cultures and were induced for 1 h with 0.5 mM IPTG. The β-galactosidase (β-gal) activity of aliquots from the culture was determined by the method of Miller (17). The OD_590_ of the cultures were noted. 100 μl cultures were removed in Z-buffer and lysed with SDS and chloroform. 200 μl ONPG was added and reactions were allowed to continue till development of yellow color. Reactions were stopped with 300 mM sodium carbonate and the color of released *ortho*-nitrophenol (ONP) and scattering were noted at 420 and 550 nm, respectively.

For statistical analysis, all data reported are the arithmetic mean (±SD) from five independent experiments performed in triplicate. The results were analyzed by one-way analysis of variance (ANOVA) followed by post hoc Tukey's Test accepting *P* < 0.001 as a level of significance. Data analyses were performed using the Prism software (GraphPad, San Diego, CA, USA).

### Circular Permutation Assay

DNA 6 was cloned at the XbaI site of the specialized vector pBend4 to get the clone pBendDA (Supplementary Table S1) and six DNA fragments for the circular permutation assay were isolated from pBendDA by digestion with BglII, XhoI, EcoRV, NcoI, SmaI and NruI, respectively. These fragments were end labeled using T4 polynucleotide kinase in the presence of [γ^32^P]-ATP using standard procedures. Gel shift assays were performed as described above; the DNA–protein complexes were separated on 8% (wt/vol) polyacrylamide gels run at 200 V for 6 h at 4°C. The magnitudes of the apparent bending were calculated from the variations in the mobilities of the DNA–protein complexes in the circular permutation analysis using the formula

(1) \begin{equation*} R_L = \cos^{\alpha }\!/_{2} \end{equation*}

where *R_L_* (relative mobility) is defined as the ratio of the minimally (*r*_min_) and maximally (*r*_max_) retarded bands. ‘*r*’ is the ratio of *μ*_bound_ and *μ*_free_. Mobility (*μ*) is in turn defined as the distance migrated from the origin. *α* is the angle by which the DNA is bent from linearity (18).

### DNA–protein docking and MD simulations

The PDB coordinates of the B-DNA with the sequence as found by DNase I footprinting were generated using 3D-DART web server (19). The DNA models were analyzed using Curves+ (20). The DNA and the crystal structure of HlyU_Vc (PDB ID: 4OOI, (15)) docking was done using Haddock server: easy interface (21). The experimentally determined active residues for both the protein and the DNA were mentioned in the submitted job.

The best docked model of DNA-HlyU_Vc complex with maximum Haddock score was chosen for MD studies. All simulations and equilibration were performed using the sander module of AMBER 10.0 software package at 300 K (22). The structure was solvated in a cuboid periodic box of explicit water with water molecules extending 6 Å outside the protein on all sides. The water molecules were described by the transferable intermolecular potential three point (TIP3P) model (23). The molecules were minimized using steepest descent for 500 cycles, followed by conjugate gradient method for 20 000 cycles prior to equilibration and dynamics run. The system was heated to 300 K within 40 ps and equilibrated following minimization. Bonds involving hydrogen were constrained with the help of the SHAKE algorithm. The production run was carried out for 20 ns, employing constant pressure periodic boundary conditions (24). A non-bonded cutoff distance was set to 12 Å and 2 fs integration time step was used. The coordinates were saved after each 2 ps. The analyses of the trajectories were performed using the ptraj module of AMBER. The figures and movies were generated using Pymol (http://www.pymol.org/) and VMD (http://www.ks.uiuc.edu/Research/vmd/), respectively. The bending of helices was computed using HELANAL-PLUS, an online server that is useful in characterizing the overall geometry of helices from its Cα atom coordinates (25).

## RESULTS

### HlyU_Vc binds to an imperfect palindrome about 164 bp upstream of hlyA transcription start site

**Figure 1.**
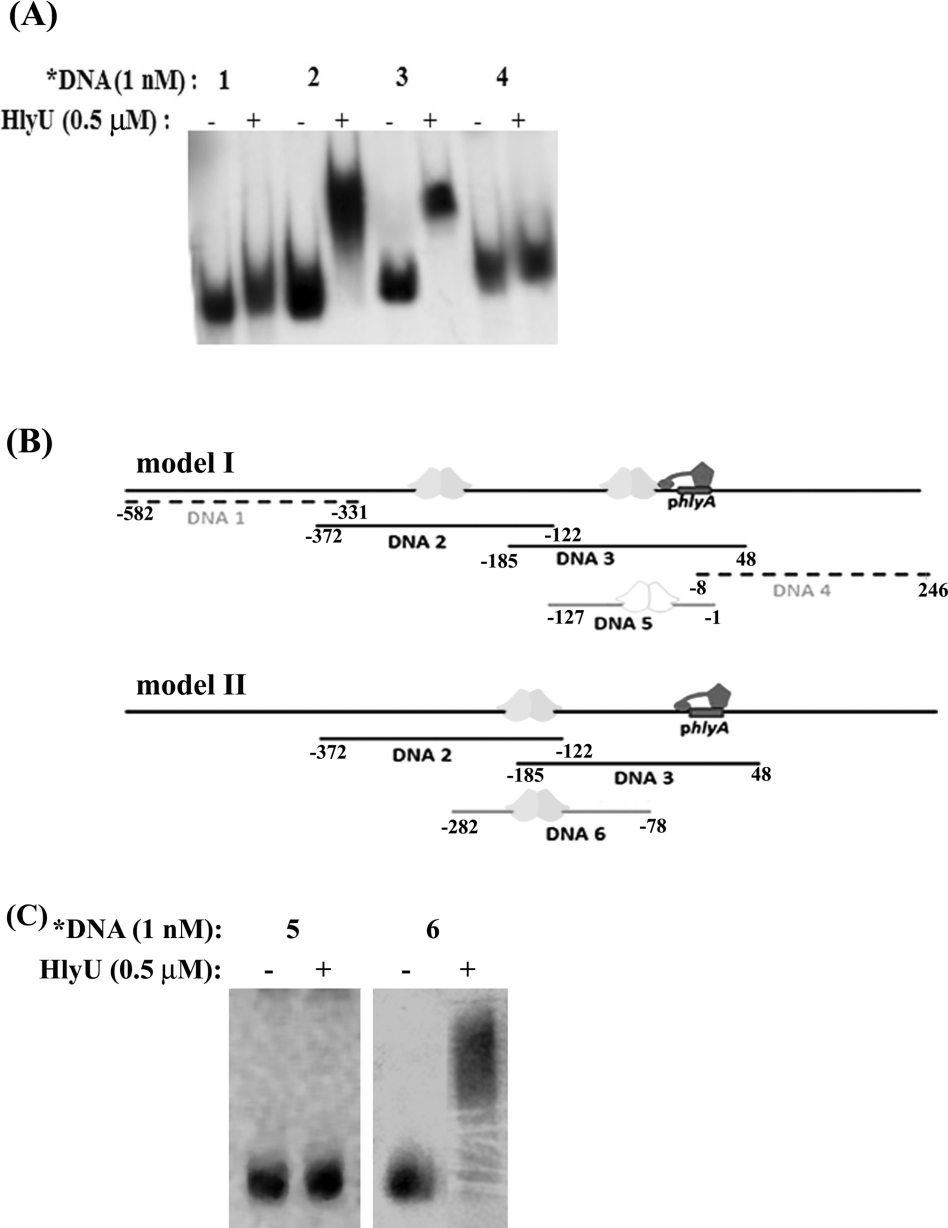
The binding of HlyU_Vc to the upstream region of the *hlyA* gene. (**A**) EMSA showing protein binding to DNA sequences 1–4 (Table 1). (**B**) Models for HlyU_Vc binding that can account for shift in mobility of both DNA 2 and DNA 3. (model I) There are separate binding sites for HlyU_Vc on both DNA 2 and DNA 3, in which case HlyU_Vc should show shift with DNA 5. (model II) HlyU_Vc binds to the overlapping regions of DNA 2 and DNA 3, in which case HlyU_Vc should show shift with DNA 6. The dimeric HlyU_Vc, *hlyA* promoter (p*hlyA*) and the RNA pol are represented as cartoons. (**C**) EMSA showing protein binding to DNA sequences 5–6.

As Williams and Manning showed a 710-bp DNA sequence upstream of *hlyA* gene in conjunction with HlyU_Vc increases HlyA production (16), we scanned the region upstream of the *hlyA* gene for the precise delineation of the HlyU_Vc binding site. Four DNA fragments (Table 1) from the region (−582 to +246 bp) spanning the *hlyA* promoter were amplified by PCR from the *V. cholerae* N16961 genomic DNA such that there is ∼50-bp overlap between consecutive fragments. EMSA revealed that HlyU_Vc binds to both DNA 2 and DNA 3 (Figure 1A). The results indicated that HlyU_Vc binding can possibly occur by either of the two ways as modeled in Figure 1B. According to model I, both the DNA fragments can show shift if there are separate binding sites for HlyU_Vc. To test this possibility, EMSA was performed with a 127-bp DNA fragment (DNA 5, Table 1); the fragment is a part of DNA 3 excluding ∼58 bp at the 5′ end that has a sequence overlap with the 3′ end of DNA 2. Since there was no complex formation with this fragment (Figure 1C), we assessed the other possibility that HlyU_Vc binds to the overlapping regions of DNA 2 and DNA 3 (model II). A 205-bp fragment (DNA 6, Table 1) with the overlap between DNA 2 and 3 residing at the center of the nucleotide string was considered. On performing gel shift assays, HlyU_Vc protein was found to bind DNA 6 (Figure 1C).

**Figure 2.**
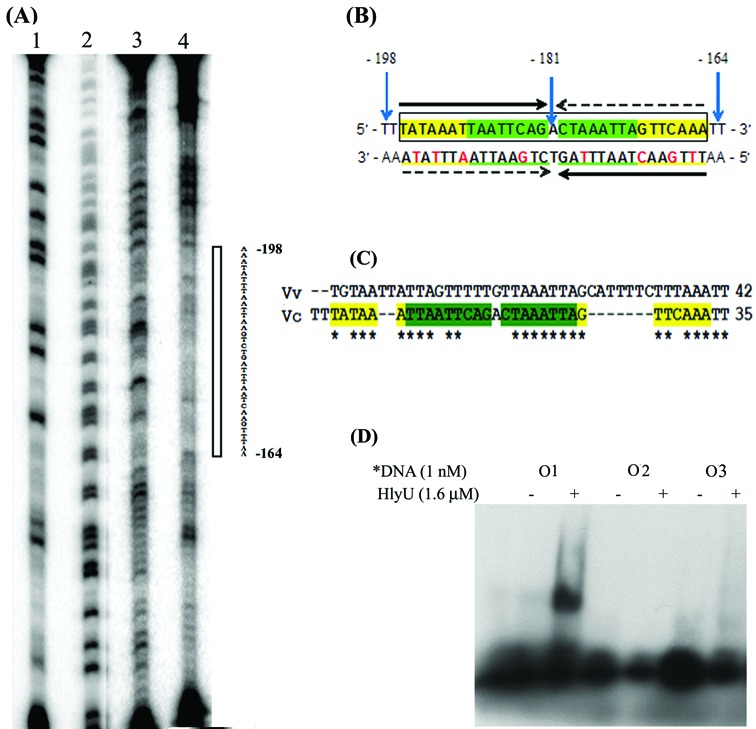
(**A**) Identification of the HlyU_Vc binding sites using DNase I footprint analysis of the antisense strand. Lane 1, G ladder; lane 2, A+G ladder; lane 3, DNase I ladder; and lane 4, DNA–protein complex digested with DNase I. The open bar indicates the protection region from 164 to 198 bp upstream of the transcription start site of *hlyA* gene. (**B**) The sequence of the double-stranded DNA derived from the footprinting experiment. The green highlighted sequence shows the internal 17-bp palindrome that can be extended by seven more bases on either side (shown in yellow). Numbers of the bases of both ends and the center are designated with respect to the transcription start site. The horizontal arrows indicate the corresponding palindromic half sites. The red colored bases on the 3′→5′ strand denote the mismatches in the palindrome. (**C**) Alignment of the DNA sequences from *V. vulnificus* (34) and *V. cholerae* that bind HlyU. (**D**) EMSA with oligonucleotides (Table 2), O1 being the original palindrome, and O2 and O3 having each of the half sites of O1.

Subjecting the HlyU_Vc binding stretch (DNA 6) to DNase I footprinting, a protection spread over 35 residues (−164 to −198 bp) was found (Figure 2A). An imperfect palindrome (5′-TAATTCAGACTAAATTA-3′), −173 to −189 bp upstream of the *hlyA* transcription start site, on which the recognition helices of HlyU_Vc sits, was identified—over the stretch of 17 bases between the complementary strands, there is only one mismatch on either side of the central position (Figure 2B). This internal 17 residue palindrome can be extended by seven residues (shown in yellow color in Figure 2B) on either side—however, it introduces three more mismatches. This additional region may be important for interaction of the wing of wHTH motif. The binding site sequences found for HlyU_Vv and HlyU_Vc were aligned (Figure 2C), which shows ∼71% base identity with common stretches of T and A. Next we performed EMSA with oligonucleotide sequences O1, O2 and O3 (Table 2). O1 is the 35-bp palindrome that binds HlyU_Vc. The 5′ end of O2 is the sequence from DNA 6 immediate upstream of O1; the sequence of O2 ends with the first half of O1—thus it retains one half of the binding stretch. O3 is an altered sequence that retains the second half of O1 while the first half is replaced by a G trail. HlyU_Vc was found to bind only to O1, while the others showed no shift (Figure 2D). Thus the individual half sites are incapable of binding and the entire palindrome is crucial in binding HlyU_Vc.

**Table 2.** Oligonucleotides (35 bp) used for binding studies

Fragment	Strand	Sequence^a^
Name	Identity	
O1	Top	5′-**TTTATAAATTAATTCA*GACTAAATTAGTTCAAATT***-3′
	Bottom	5′-AATTTGAACTAATTTAGTCTGAATTAATTTATAAA-3′
O2	Top	5′-ATTAATCTTTAAATCAAC**TTTATAAATTAATTCAG**-3′
	Bottom	5′-CTGAATTAATTTATAAAGTTGATTTAAAGATTAAT-3′
O3	Top	5′-TTGGGGGGGGGGGGGG***GACTAAATTAGTTCAAATT***-3′
	Bottom	5′-AATTTGAACTAATTTAGTCCCCCCCCCCCCCCCAA-3′

^a^The original palindrome (whole or in part) in the top strand is shown in bold; italics font type is used to distinguish its second half.

We also checked the effect on DNA binding of a few nucleotides in the palindrome. dT(-190), dA(-187), dC(-180) and dT(-175) were mutated, and were found to have no affect (data not shown). Comparing the binding sites for HlyU in different *Vibrio* species, it is interesting to note that there is no strict consensus binding site of HlyU for the transcription of different genes (26,27). Considering HlyU as a global regulator (12), it can be expected that there would be flexibility in the binding sequence and, therefore, single mutations are less expected to affect binding.

### Dimerization: a prerequisite for DNA binding by HlyU_Vc

**Figure 3.**
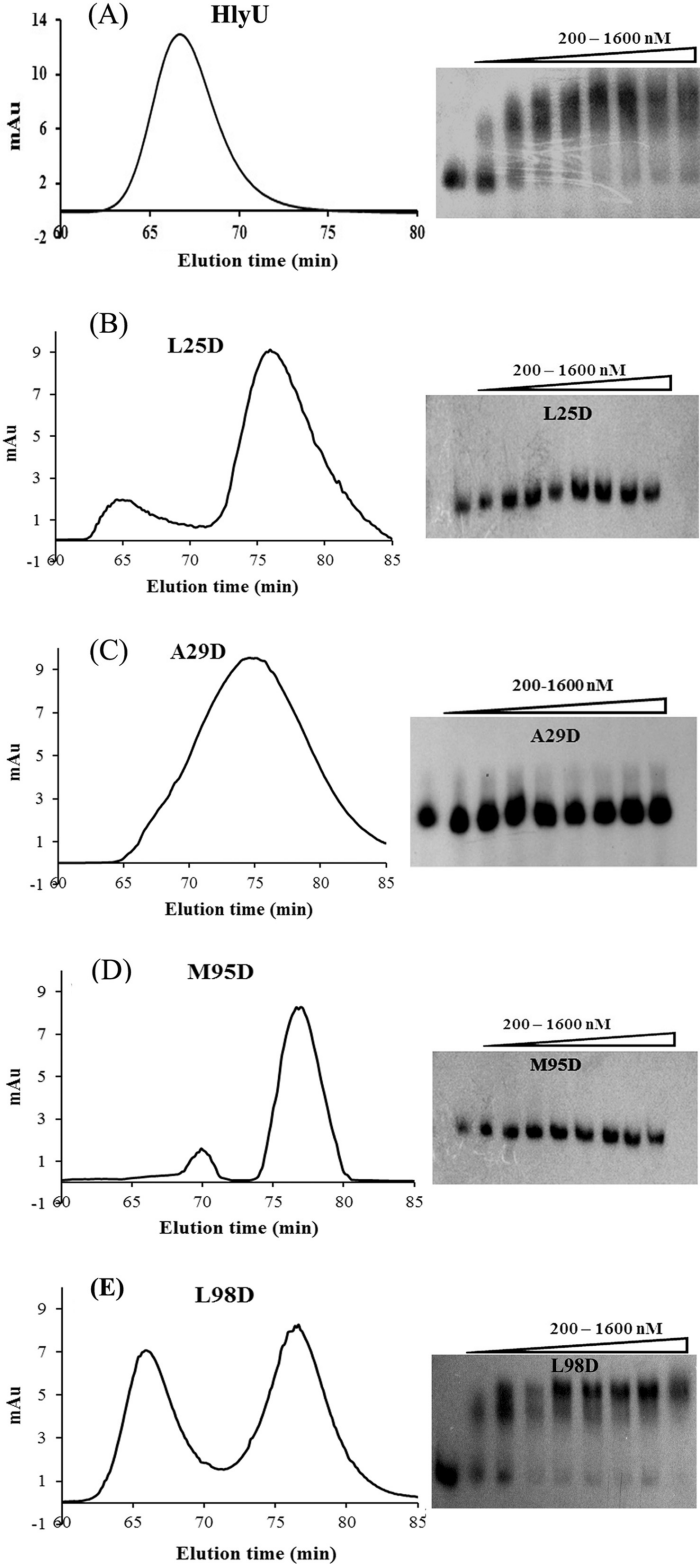
Profiles of gel filtration chromatography and EMSA of (**A**) HlyU, and the self-contact residue mutants, (**B**) L25D, (**C**) A29D, (**D**) M95D and (**E**) L98D. The leftmost lane for each gel is the free probe (DNA6, Table 1). All the proteins have been used in the concentration range, 200–1600 nM, with 200-nM increment.

In 2-fold symmetry-related protein subunits, a residue close to the 2-fold axis may interact with the same residue from the other subunit thus making up a pair of ‘self-contacting’ residues which may be important in defining the homodimeric interface (28). Analysis of the dimeric interface (using ProFace (29)) of the crystal structure of HlyU_Vc revealed the presence of four self-contact residues: Leu25, Ala29, Met95 and Leu98 (Supplementary Figures S2 and S3) (16). These hydrophobic residues were mutated to a charged residue, Asp, to induce charge–charge repulsion, consequently preventing protein dimerization. The ultimate target was to emphasize the importance of protein dimerization on DNA binding. Size exclusion chromatography of the proteins indicated that while the wild-type protein eluted entirely as dimer (Figure 3A), L25D, A29D and M95D showed predominant monomer population (Figure 3B–D). Interestingly, L98D mutation affected dimerization to a lesser extent with both the dimer and monomer being present in nearly equal amounts (Figure 3E). In accordance to the oligomerization behavior, L25D, A29D and M95D did not bind DNA (Figure 3B–D), whereas in the case of L98D, there was considerable DNA binding (Figure 3E).

At even higher concentrations of the proteins, L25D, A29D and M95D failed to bind DNA (Supplementary Figure S4A). However, L98D showed a similar DNA binding profile as wild-type HlyU_Vc in the same high range of protein concentrations. Among the four self-contact residues, Leu98 is the only one that lies at the rim of the dimeric interface (15); the others lie in the core region and therefore influence the dimeric association more strongly (30). Moreover, Leu98 makes fewer contacts than the others (Supplementary Figure S2), lying close to the protein exterior (Supplementary Figure S3). Therefore, a mutation in this residue is less likely to alter the oligomeric status of HlyU as compared to other self-contact mutants.

### Amino acids essential for DNA-HlyU_Vc binding

**Figure 4.**
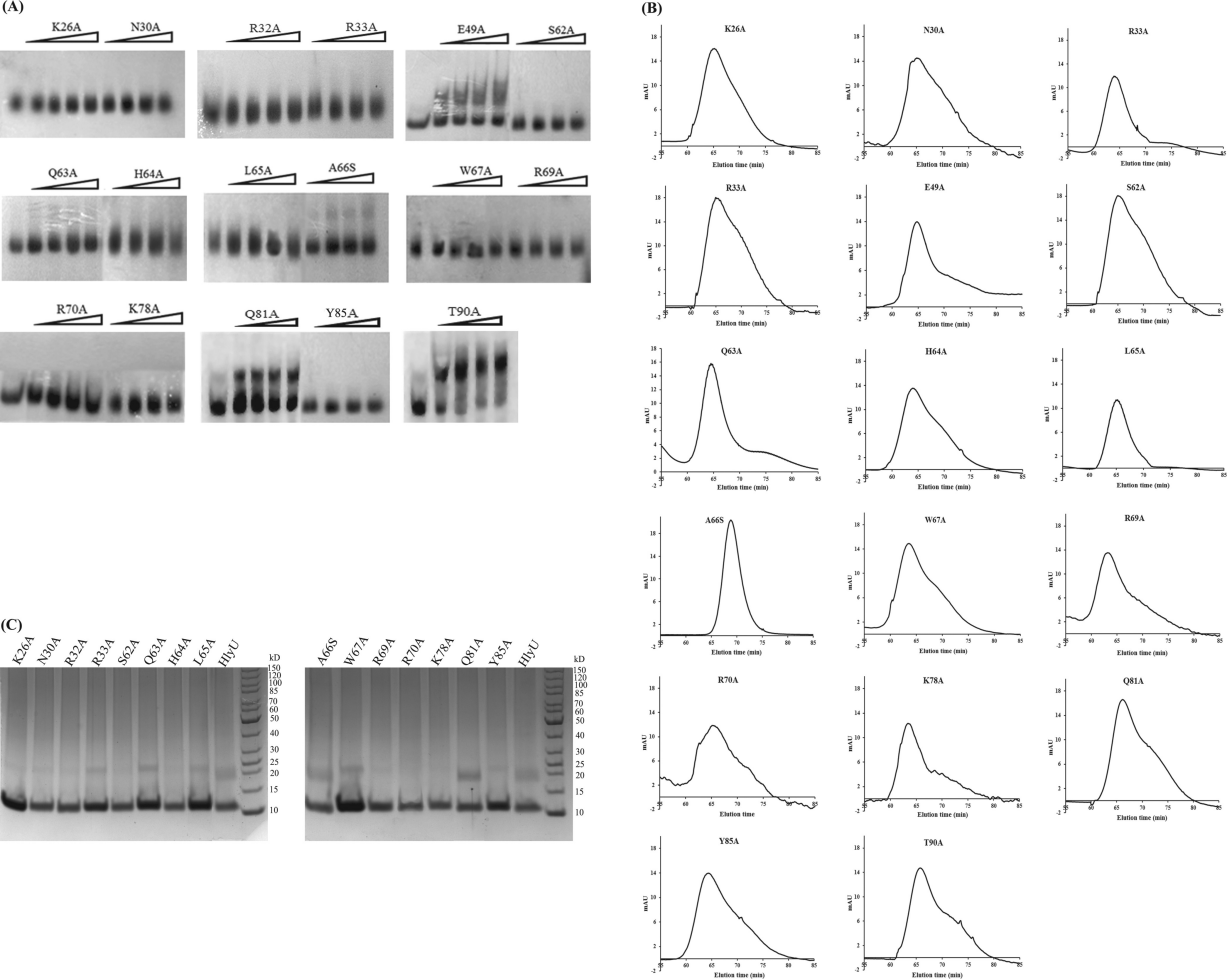
(**A**) EMSA for binding of HlyU_Vc mutants to the *hlyA* regulatory region. The leftmost lane for each gel contains the free probe (DNA6, Table 1). All the proteins have been used in the concentration range, 200–1600 nM, with 200 nM increment. Gel filtration profiles (**B**) and SDS-PAGE (**C**) of mutants used in (A).

Based on the crystal structure of HlyU_Vc (PDB ID: 4OOI, (15)), *in silico* model of HlyU_Vc -DNA complex (14) and DNA binding studies on HlyU from *Vibrio vulnificus* (HlyU_Vv) (31), we predicted some amino acids which may be essential for HlyU_Vc's biological function. The amino acids chosen were Lys26 from the α1 helix, Asn30 from the loop between α1 and α2 and Arg32, and Arg33 from the α2, Ser62, Gln63, His64, Leu65, Ala66, Trp67, Arg69 and Arg70 from α4, Lys78 from β2, Gln81 from the turn, and Tyr85 from β3. In addition, we selected some residues away from the DNA binding sites as negative controls, Glu49 from the α3 and Thr90 from α5. Ala was converted to Ser and all others were mutated to Ala to understand their functional significance. K26A, N30A, R32A, R33A, S62A, Q63A, H64A, L65A, W67A, R69A, R70A, K78A and Y85A mutants did not show any DNA binding at the protein concentrations tested (Figure 4A). The control mutations E49A and T90A did not affect DNA binding. A66S showed very slight DNA binding and unexpectedly, Q81A exhibited considerable shift. All the mutations mentioned above did not impair the dimerization of the protein (Figure 4B). The mutants were neither partially degraded nor did they behave differently from the wild-type protein on SDS-polyacrylamide gel electrophoresis (PAGE) (Figure 4C). Although the mutants were shown to be incapable of DNA binding (Figure 4A), we wanted to see their behavior at higher protein concentrations (Supplementary Figure S4B). While N30A, R32A, R33A, Q63A, R69A and R70A mutants were absolutely defective in DNA binding, K26A, W67A and Y85A produced some DNA–protein complexes. Like HlyU_Vc at higher concentrations, the other mutants (S62A, H64A, K78A, Q81A) produced supershifted bands, as also the control mutant T90A.

### DNA-HlyU_Vc interaction enhances promoter activity

**Figure 5.**
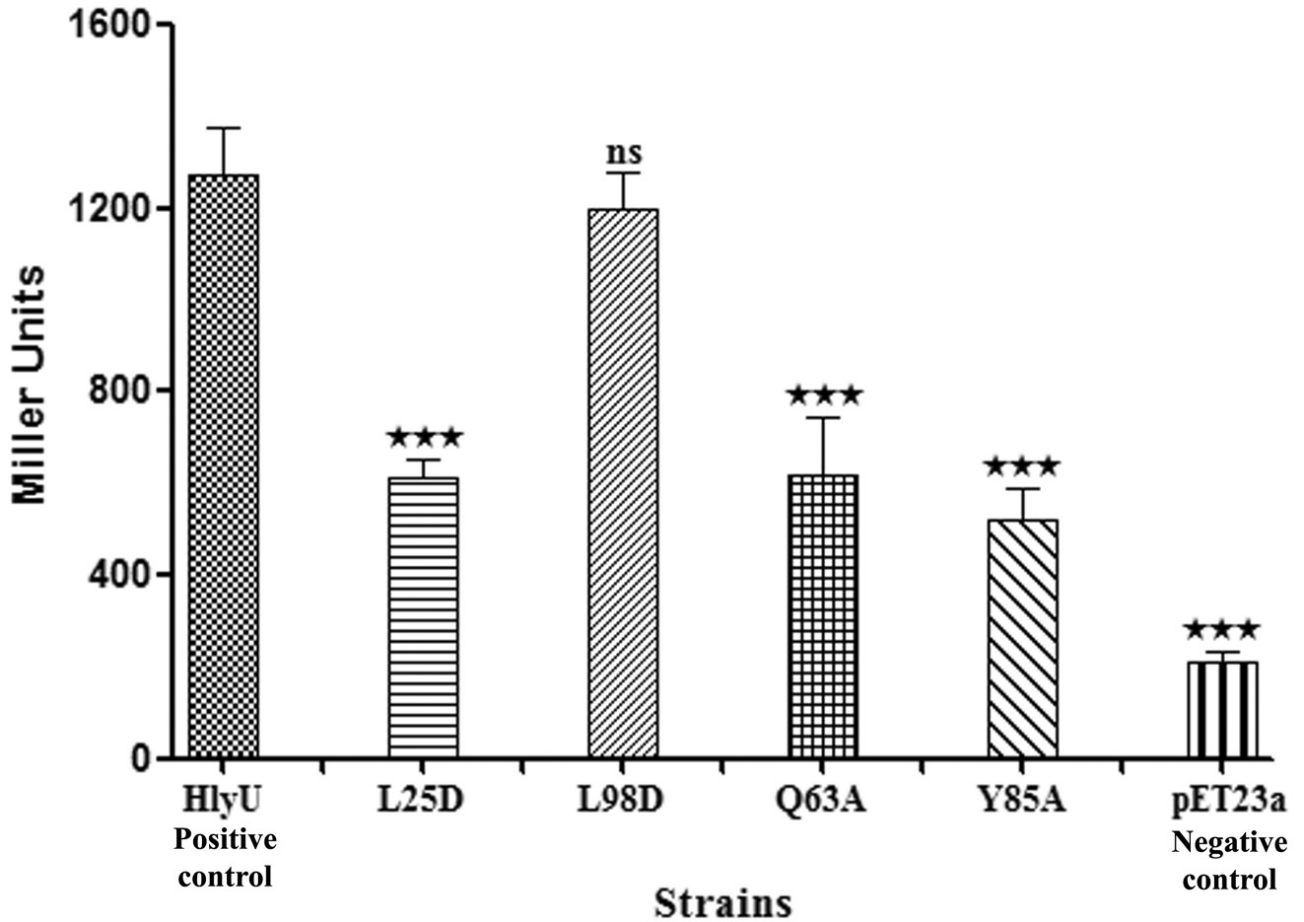
Comparison of HlyU_Vc regulatory function by β-galactosidase activities of constructs with the *hlyA* promoter/enhancer-*lacZ* fusion. HlyU_Vc wild-type protein is the positive control while the blank plasmid (pET23a) is the negative control. Two dimerization mutants (L25D and L98D) and two DNA-binding mutants (Q63A and Y85A) have also been assessed. All data reported are the arithmetic mean (±SD) from five independent experiments performed in triplicate. The results were analyzed by one-way ANOVA followed by post hoc Tukey's Test accepting *P* < 0.001 as a level of significance (indicated by *** on top of the bars).

The ability of HlyU_Vc to activate transcription, *in vivo*, was monitored by β-gal assay. A plasmid containing *lacZ* gene under the control of the *hlyA* promoter along with the HlyU_Vc binding site and a clone of *hlyU_Vc* gene were co-transformed in *E. coli* BL21 (DE3). Wild-type HlyU_Vc significantly boosted transcription of the reporter gene as compared to the negative control (pET23a) (Figure 5). Y85A, Q63A and L25D mutants failed to physically interact with DNA, and when compared to wild-type HlyU_Vc showed much less activity, which was statistically significant at *P* < 0.001. The relative activity found for the positive control in the reporter gene assay, thus, demonstrates the *in vivo* binding of HlyU_Vc to the identified DNA partner and the positive control exerted by HlyU_Vc on the *hlyA* promoter. The significantly different activity levels for the mutation in the dimerization interface (L25D), recognition helix (Q63A) and in the wing (Y85A) as compared to the wild-type activity emphasize the importance of these regions in the biological functioning of HlyU_Vc. β-gal activity comparable to wild-type HlyU_Vc was obtained for L98D (the difference being statistically non-significant at *P* < 0.001). This is in agreement with the finding that this mutant retains the dimeric structure to a considerable extent and binds DNA *in vitro* (Figure 3E).

### DNA-HlyU_Vc interactions induce bending of DNA

**Figure 6.**
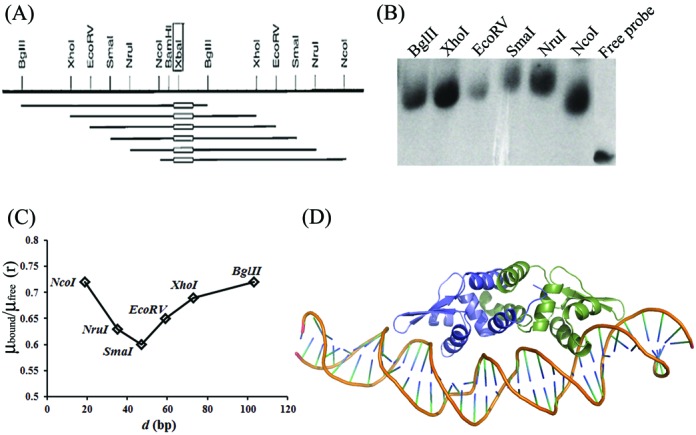
(**A**) Restriction map of the probes used in the DNA bending assay (from vector pBend4); the oblong indicates location of the DNA 6 in the DNA probe. (**B**) Circular permutation assay to determine deviation from linearity of DNA on binding to HlyU_Vc. (**C**) Plot of *μ*_bound_/*μ*_free_ (‘*r*’, the ratio of mobility (*μ*) of the bound form and the free probe for each restriction enzyme digested fragment with the DNA6 insert) versus the position (*d*, in bp) of the DNA insert from the 5′ end of each restriction fragments. The minimum and maximum values of the ratio were used to calculate α (bend angle) according to Equation (1). (**D**) DNA-HlyU_Vc model after 10 ns MD simulation.

DNA-binding proteins often use combination of base readout and shape readout mechanisms to bind to specific sites. Shape readouts may be results of intrinsic conformation of the nucleic acid or the structural changes brought upon DNA–protein interaction. The circular permutation assay, used to decipher any structural change of the double helix on DNA-HlyU_Vc complex formation, indicated that the angle of divergence of DNA 6 from linearity is 68 ± 2° (Figure 6A–C).

### MD simulations emphasize the solution studies

Models of the 35-bp partner DNA and HlyU_Vc were generated with different bent angles of the DNA. However, the best results were obtained for the complex with 68° bend and were used for MD simulations for 10 ns. The complex retains a bend angle of ∼55° after the simulation (Figure 6D), which is consistent with the finding that HlyU_Vc binding indeed induces considerable curvature in the DNA (Supplementary Movie SM1).

DNA binding also induces some conformational changes in the protein structure. Noticeable change occurs in α1 whose geometry changes from being kinked (unbound state) to curved (bound state) with a significant decrease in the value of bend angle (Supplementary Figure S5). In α4, there is a 2-fold increase in helix bending on DNA binding; the opposite being the case with α5.

**Figure 7.**
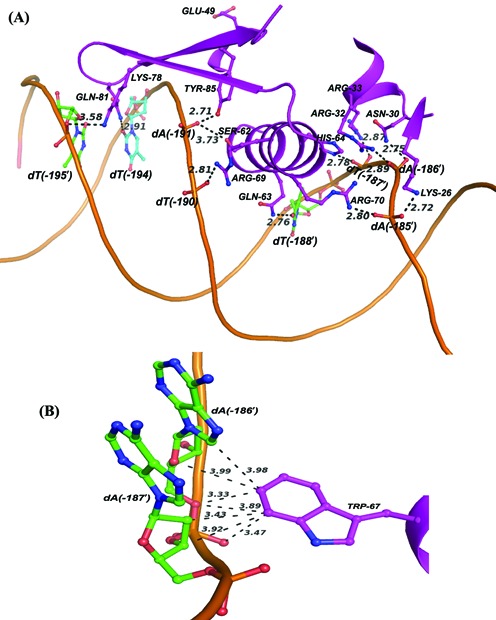
Model showing the interaction of HlyU_Vc with partner DNA as obtained from MD simulations. Interactions between (**A**) the wHTH motif and DNA, and (**B**) Trp67 and DNA bases indicating distances <4 Å. The interactions are shown only with respect to subunit A of HlyU_Vc.

A model showing the putative interactions of the functionally important amino acids with the DNA is shown in Figure 7. Among the residues that were experimentally found to be important in DNA binding, the side chains of Lys26, Asn30, Arg32, Arg33, Ser62, Gln63, His64, Arg69, Arg70, Lys78 and Tyr85 point toward the DNA bases and simulations indicate the existence of hydrogen bonding (Supplementary Table S2 and Figure 7A) or close van der Waal's contact involving Trp67 (Figure 7B). Two non-polar residues, Leu65 and Ala66, whose mutations resulted in altered DNA binding, may have an indirect effect by changing the stability of the recognition helix in which they are located.

The percentage occupancy of the hydrogen bonds between the amino acids and the corresponding bases during MD simulations (Supplementary Table S2) may explain the dependence of mobility shift on protein concentration (Figure 4A and Supplementary Figure S4B). If we classify the strength of DNA binding into three groups, ‘strong’ (hydrogen bond occupancy of >15%), ‘medium’ (10–15%) and ‘weak’ (<10%), mutants of “strong” residues (Asn30, Arg32, Arg33, Gln63, Arg69 and Arg70) would be absolutely non-DNA binding irrespective of protein concentration. The mutants of ‘medium’ residues (Lys26, Trp67 and Tyr85) showed DNA binding only at high protein concentrations. For the last category (Ser62, His64, Lys78 and Gln81), though there may be some binding at low concentrations, all show supershift at high concentrations.

**Figure 8.**
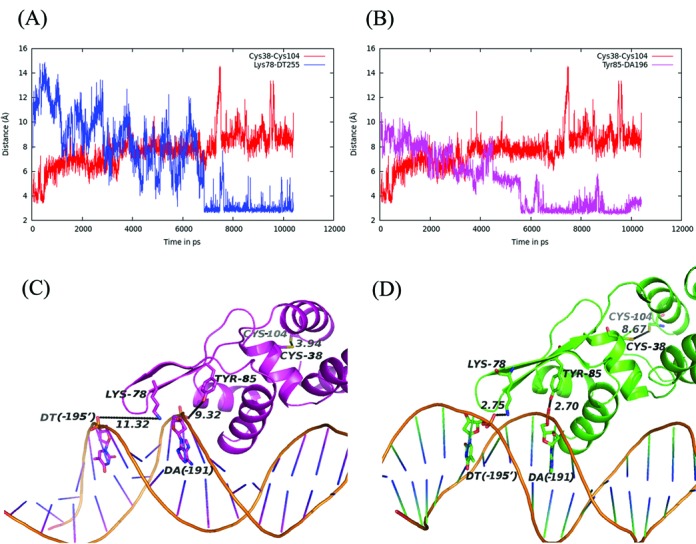
Correlation of the distances between Cys38 and Cys104 with those between (**A**) Lys78 and DNA (dT -195′), and (**B**) Tyr85 and DNA (dA -191) during the simulation; structures are displayed showing these distances at the (**C**) early phase of simulation and (**D**) at the end. The numbering of the base is shown in Figure 2B.

Another interesting observation in the MD simulation trajectory is the relation of the distance between the two Cys residues in the structure (at 38 and 104) and the contacts important in DNA binding. During the course of simulation there was not much visible change in the interaction between the major groove and the recognition helix. However, the interaction of the wing with the DNA seemed to be quite dynamic. Therefore, two very important wing residues, Lys78 and Tyr85, were chosen to highlight their mode of DNA contact. It was found that these residues moved away from the partner bases when the distance between Cys38 and Cys104 was small and vice versa (Figure 8).

Simulation of the unbound HlyU_Vc exhibited a reduction in the Cys38-Cys104 distance. The structure from the trajectory with the shortest Cys38-Cys104 distance (∼3 Å) indicated a greater distance between the wing and the recognition helix (∼17 Å). In contrast, in the DNA bound form, the distance between Cys38-Cys104 showed an increase in the value during simulation (Supplementary Movies SM2 and SM3). Also the wing is closer to the recognition helix (by ∼3 Å) (Supplementary Figure S6), allowing generous contacts with both the grooves of the DNA.

## DISCUSSION

To have a complete picture of a gene expression system, it is of primary importance to elucidate the regulatory sequences that include the promoter region together with enhancer elements to which activator proteins bind. HlyU is an important virulence activator, responsible for the activation of the *rtxA1* operon in *V. vulnificus*, the *plp-vah1* gene cluster and the *rtxACHBDE* gene cluster in *Vibrio anguillarum*, and *hlyA* gene and probably other virulence factors in *V. cholerae* (15). Recent investigations by several groups have thrown light on regulation of virulence in *V. vulnificus* and *V. anguillarum* by HlyU (27, 32). As far as the *V. cholerae* system is concerned, the promoter sequence for *hlyA* gene had been determined by Williams and Manning in the 1990s (16). They also identified HlyU_Vc to play some role in *hlyA* gene activation. However, at this time there is a lack of information on specific interaction between HlyU_Vc and the enhancer sequence in the *hlyA* expression system.

In this article, HlyU_Vc has been demonstrated to specifically bind to a 17-bp imperfect palindrome 5′-TAATTCAGACTAAATTA-3′ 173 bp upstream of the *hlyA* transcription start site (Figure 2). Studies with the mutations at the self-contacting interface residues, Leu25, Ala29, Met95 and Leu98, indicated that homodimerization of the protein is an essential criterion for DNA binding (Figure 3). From the crystal structure of HlyU_Vc (PDB ID: 4OOI, (15); Supplementary Figure S1), the residues constituting the wHTH motif were clearly deciphered. Lys26, Asn30, Arg32, Arg33, Ser62, Gln63, His64, Leu65, Trp67, Arg69, Arg70, Lys78 and Tyr85 residues were found to be indispensable for DNA binding *in vitro* (Figure 4), as also suggested by the structure. The reporter gene assay strongly suggests that DNA–HlyU_Vc interaction augments the promoter activity while the mutated proteins (Y85A, Q63A and L25D) fail to do so (Figure 5). The protein binding induced the target DNA to deviate 68 ± 2° from linearity (Figure 6). This is in conformity with the protein structure which indicates a distance less than 34 Å between the two recognition helices in the dimer (15), necessitating a bent DNA partner. On docking, the protein fits perfectly with the α4 helices and the wings in the major and minor grooves, respectively, of a significantly bent DNA (Figure 6D and Supplementary Movie SM1). wHTH proteins are known to interact with DNA with the recognition helices sitting on the major grooves and the wings drooping over the minor grooves (33). In our case, the involvement of both the types of grooves was also ascertained by competition with groove-specific dyes, the details of which are available as Supplementary Text S1. MD simulation studies validate the contacts of the DNA phosphates/bases with the experimentally determined important amino acids of HlyU_Vc (Supplementary Table S2).

Our current findings open up many more questions about the molecular mechanism of regulation by HlyU_Vc. Prokaryotic transcription control may involve direct contact of regulatory proteins (bound to DNA adjacent to the target promoter) with RNA polymerase, or alternatively, the regulatory proteins bound at more distally located enhancer sites may activate the promoter by DNA-looping, or with the help of mediator proteins, or by anti-repression (34). HlyU from *V. vulnificus* and *V. anguillarum* has been shown to act as an anti-repressor relieving the repression by H-NS (26,35). Liu and Crosa have proposed a model where the regulatory DNA is looped and involved in a bridge with H-NS that represses the *rtx* gene expression (36). Our results indicate the HlyU_Vc binding site to be quite distant from the promoter which compels us to reflect on the possible mode of DNA looping with involvement of accessory mediator or repressor proteins. It is likely to observe such H-NS-mediated anti-repression in HlyU_Vc too (Pal,A. *et al.*, unpublished data).

**Figure 9.**
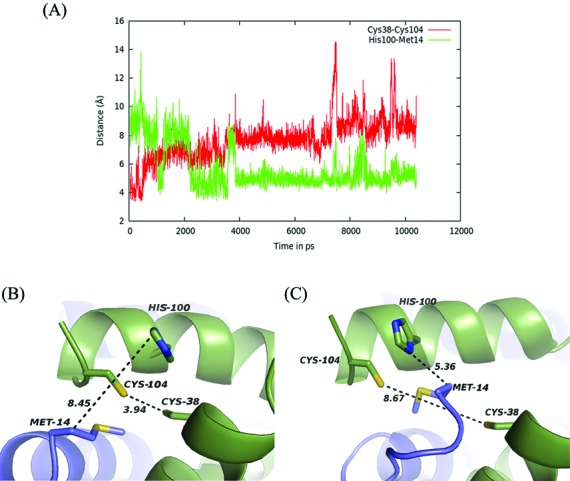
(**A**) Correlation of the distance between Cys38 and Cys104 of chain A with that involving Met14 (chain B) and His100 (chain A) during simulation; structure are displayed showing these distances at the (**B**) early phase of simulation and (**C**) at the end.

Virulence genes are usually conditionally expressed under specific stimuli and are likely to be downregulated by some other. *V. cholerae* is able to respond to host environmental signals by activating transcriptional regulatory cascades. However, the mode of functioning switch in HlyU family of proteins for regulating the virulence determinants is yet unexplored. While several SmtB/ArsR family proteins use metal coordination for transformation into DNA non-binding form, many wHTH proteins of related structures, especially BigR (29% sequence identity to HlyU_Vc (www.ebi.ac.uk/Tools/msa/clustalw2/)), while 174 residues out of 187 (in dimer) structurally aligned ((www.ccp4.ac.uk/); Supplementary Figure S8A and B) have been found to undergo DNA-binding/release using a redox switch. In the DNA binding, reduced thiol form of BigR, the two Cys residues are wide apart (>9 Å); however, a C-terminal Tyr and an N-terminal Met residue from the two different subunits come closer as if to disrupt the formation of the disulphide bond (Supplementary Figure S8C). The oxidized BigR structure indicates increased distance between the Tyr and the Met residues (Supplementary Figure S8D) (37). The MD simulations suggest the possibility of a similar redox switch in HlyU. Simulation of the unbound HlyU_Vc indicated a negative correlation between two distances—one between Cys38 and Cys104 and the other involving Met14 and His100, all of these residues occupying the equivalent positions of the redox switch protagonists in BigR. The trajectory of the DNA bound HlyU_Vc revealed a steady concomitant decrease in the distance between Met14 and His100 with increasing stabilization of the DNA–protein complex and increasing distance between the Cys residues (Figure 9 and Supplementary Movie SM2). The closing of the wings onto the minor groove with the parallel distancing of the Cys residues, thus, leading to a better complex interaction, points toward a plausible analogy to the BigR redox model. Therefore, it would be interesting to know whether the cysteine(s) of the non-metal binding HlyU_Vc play any such redox role. It is worth mentioning here that under oxygen-limiting conditions, an environment similar to the host intestines, *V. cholerae* virulence genes are highly expressed and the pathogen may use a thiol-based switch mechanism to sense oxygen-rich aquatic environments and oxygen-limited human host signals (38–40). Anaerobiosis is indeed reported to increase HlyA production in *E. coli* and *Bacillus cereus* (41,42). It may also be added that conservation of the residues proposed to be important in the probable redox switch of HlyU_Vc is noted in several other species of *Vibrio* which share considerable sequence identity; HlyU from yet other species having divergent sequence show absence of Cys and the accessory residues (Supplementary Figure S9). With our ongoing research, we look forward to answering the yet unresolved questions, dissect the mechanistic details of regulation of *hlyA* gene by HlyU_Vc and elucidate any possible conservation in the functioning of this family of protein.

## SUPPLEMENTARY DATA

Supplementary Data are available at NAR Online.

SUPPLEMENTARY DATA
