# Supplementary material for: Identification of the target DNA sequence and characterization of DNA binding features of HlyU, and suggestion of a redox switch for hlyA expression in the human pathogen Vibrio cholerae from in silico studies
Source: Nucleic Acids Res. 2015 Jan 20;43(3):1407–17. doi: 10.1093/nar/gku1319 (PMC4330345; doi:10.1093/nar/gku1319)
Supplement: SUPPLEMENTARY DATA [file supp_43_3_1407__index.html]

Identification of the target DNA sequence and characterization of DNA binding features of HlyU, and suggestion of a redox switch for hlyA expression in the human pathogen Vibrio cholerae from in silico studies — SUPPLEMENTARY DATA 

# Identification of the target DNA sequence and characterization of DNA binding features of HlyU, and suggestion of a redox switch for *hlyA* expression in the human pathogen *Vibrio cholerae* from *in silico* studies

## SUPPLEMENTARY DATA

**Files in this Data Supplement:**

- SUPPLEMENTARY DATA
- Video SM1
- Video SM2
- Video SM3
