# Supplementary material for: Identification of the target DNA sequence and characterization of DNA binding features of HlyU, and suggestion of a redox switch for hlyA expression in the human pathogen Vibrio cholerae from in silico studies
Source: Nucleic Acids Res. 2015 Jan 20;43(3):1407–17. doi: 10.1093/nar/gku1319 (PMC4330345; doi:10.1093/nar/gku1319)
Supplement: SUPPLEMENTARY DATA [file supp_gku1319_nar-02360-v-2014-File013.doc]

**Identification of the target DNA sequence and characterization of DNA binding features of HlyU, and suggestion of a redox switch for *hlyA* expression in the human pathogen *Vibrio cholerae* from *in silico* studies**

Debadrita Mukherjee, Aritrika Pal, Devlina Chakravarty, Pinak Chakrabarti

**Supplementary Material**

Text S1. Experiments showing the contact of HlyU_Vc with the major and the minor grooves of the double helix

The major groove binding molecule, methyl green, ([7-(dimethylamino)-4-nitrophenothiazin-3-ylidene]-dimethylazanium chloride), is extensively used to study the interaction of protein at the major groove of the DNA, while Hoechst 33258, (p-(5-(5-(4-methyl-1-piperazinyl)-1H-2-benzimidazolyl)-1H-2-benzimidazolyl) phenol trihydrochloride), the minor groove binder, is used to examine the minor groove interaction . Methyl green and Hoechst 33258 were used to determine the groove interaction of the DNA-HlyU_Vc complex. The target DNA and HlyU_Vc were incubated along with either dye in increasing concentrations and gel shift assays were performed.

Both the dyes were used in excess molar concentration with respect to the protein to assure successful competition against the protein molecule for DNA binding and to get a perception about the groove interaction of HlyU_Vc. 5 fold excess of methyl green was found to displace the DNA-HlyU_Vc complex with nearly complete dissociation at 100 fold molar excess (Figure S7A). A much lower concentration of Hoechst 33258 was required to dislodge the protein from the bound DNA with complete displacement at 1:10 molar concentrations (Figure S7B).

Table S1. Strains and plasmids used in the study

| Strains or plasmids | Features |
| --- | --- |
| Strains | |
| BL21 | Used as an expression host for HlyU_Vc and the mutant proteins |
| XL1- Blue | Tet^R^  Used for the maintenance and for the regeneration of the clones |
| Plasmids | |
| pET28a-HlyU_Vc  pET28a- XnY | Kan^R^  Used for expression of HlyU_Vc and its mutants. |
| pET23a- HlyU_Vc  pET23a-XnY | Amp^R^  Bears the *hlyU_Vc* gene under the control of T7 promoter.  Used for β- galactosidase assay |
| pDA1a | Kan^R^  Bears the *lacZ* gene under the control of *Vibrio cholerae* hemolysin promoter/regulator. Vector backbone pSD5B (Jain *et al*., 1997), Used for β- galactosidase assay |
| pBend4 | Amp^R^  DNA-bending vector which was used to generate probes having the binding site in circular permutations |
| pBendDA | Amp^R^  DNA6 with XbaI overhangs cloned in pBend4 at the XbaI site. |

^R^ Denotes the antibiotic resistance

XnY Denotes the mutant protein where “X” is the residue name, “n” is the residue number of X in HlyU and “Y” is the residue to which X has been mutated.

Table S2. Hydrogen bond occupancy along the MD trajectory of the amino acids (donor) that are experimentally found to be important in DNA binding

| **Donor** | **Acceptor^a^** | **Occupancy (%)** |
| --- | --- | --- |
| Lys26-side | dA(-185′)-Side | 15.00 |
| Asn30-side | dA(-186′)-Side | 51.30 |
| Arg32-side | dT(-187′)-Side | 36.10 |
| Arg33-side | dA(-186′)-Side | 15.55 |
| Ser62-side | dA(-191)-Side | 2.99 |
| Gln63-side | dT(-188′)-Side | 17.02 |
| His64-side | dT(-187′)-Side | 4.63 |
| Arg69-side | dT(-190)-Side | 20.40 |
| Arg70-side | dA(-185′)-Side | 20.54 |
| Lys78-side | dT(-195′)-Side | 9.21 |
| Gln81-side | dT(-194)-Side | 6.97 |
| Tyr85-side | dA(-191)-Side | 14.35 |

Hydrogen bonds, where these amino acids act as acceptors, have not been shown. The interactions are shown only with respect to subunit A of HlyU_Vc.

^a^ Side corresponds to either phosphate or base. The numbering of the base (as also shown in Figure 2B) corresponds to the position upstream of the transcriptional start site; the two strands, 3′→5′ and 5′→3′, are distinguished by primed and unprimed numbers respectively.


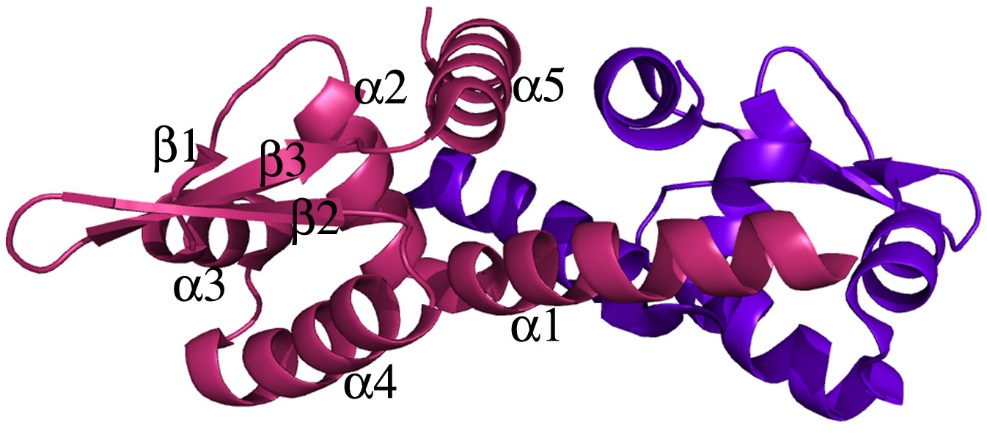


Figure S1. Overall structure of HlyU_Vc (using the A-B dimer of the crystal structure, PDB ID: 4OOI). Helices and strands are labelled in one subunit.


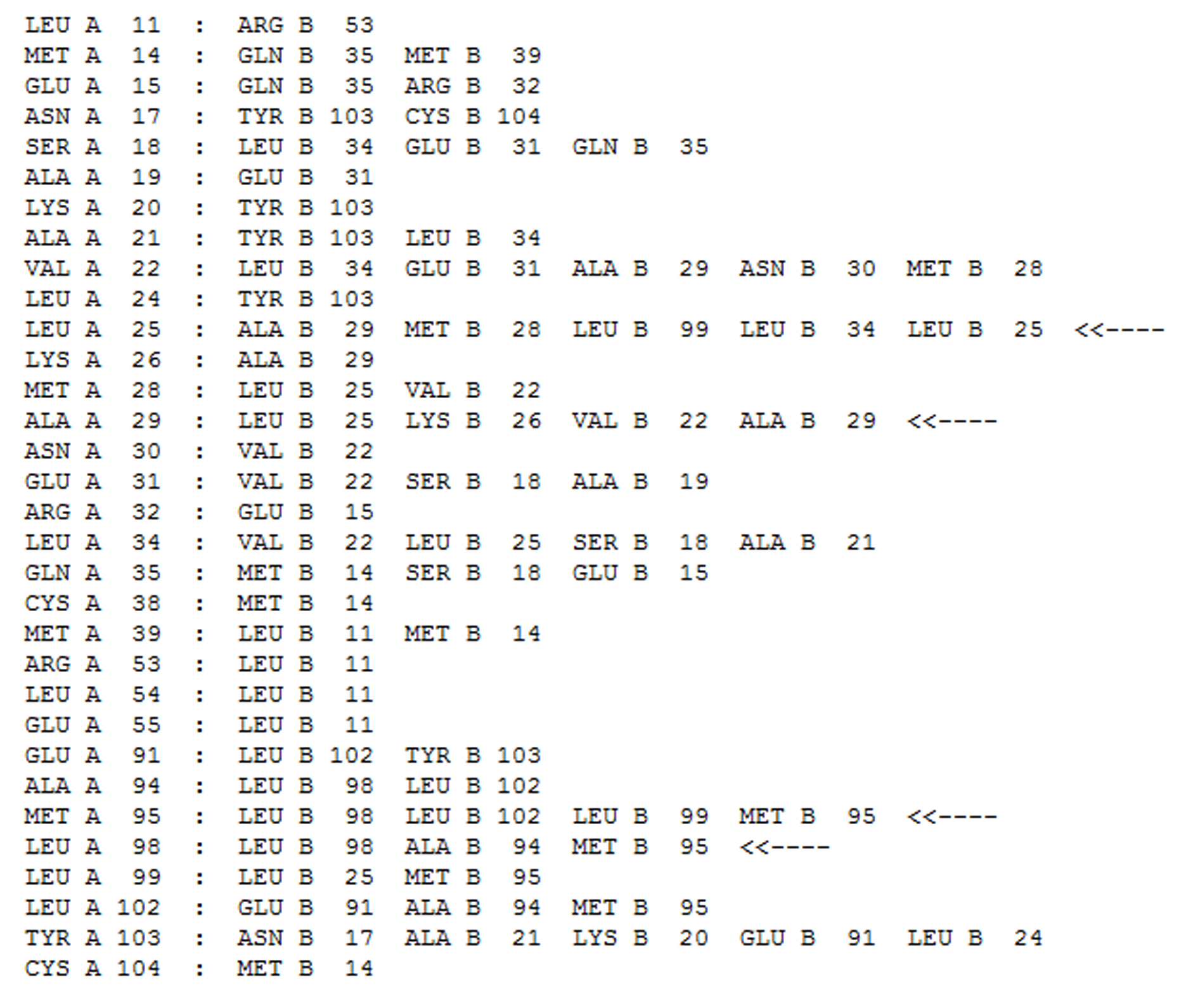


Figure S2. Output of the contact list of dimeric interface of HlyU (PDB id: 4OOI, considering subunits A and B, and as determined using ProFace . For a given component, list of neighbouring residues (within 4.5 Å) from the other component is given. Lines showing self-contact are marked by the symbol <<---- at the end.


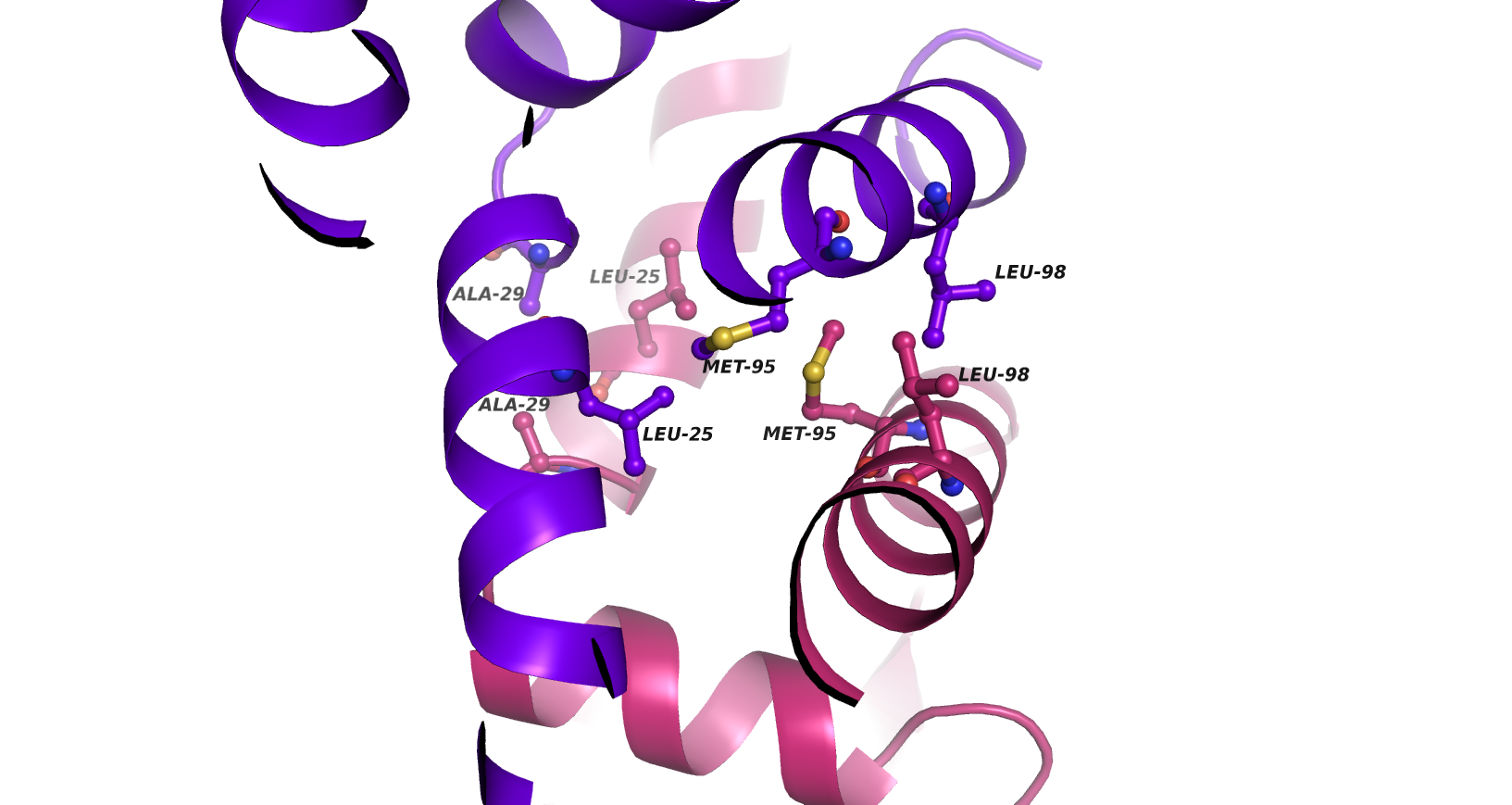


Figure S3. Self-contacting residues involving A-B subunits of HlyU_Vc.

**(A)**

**
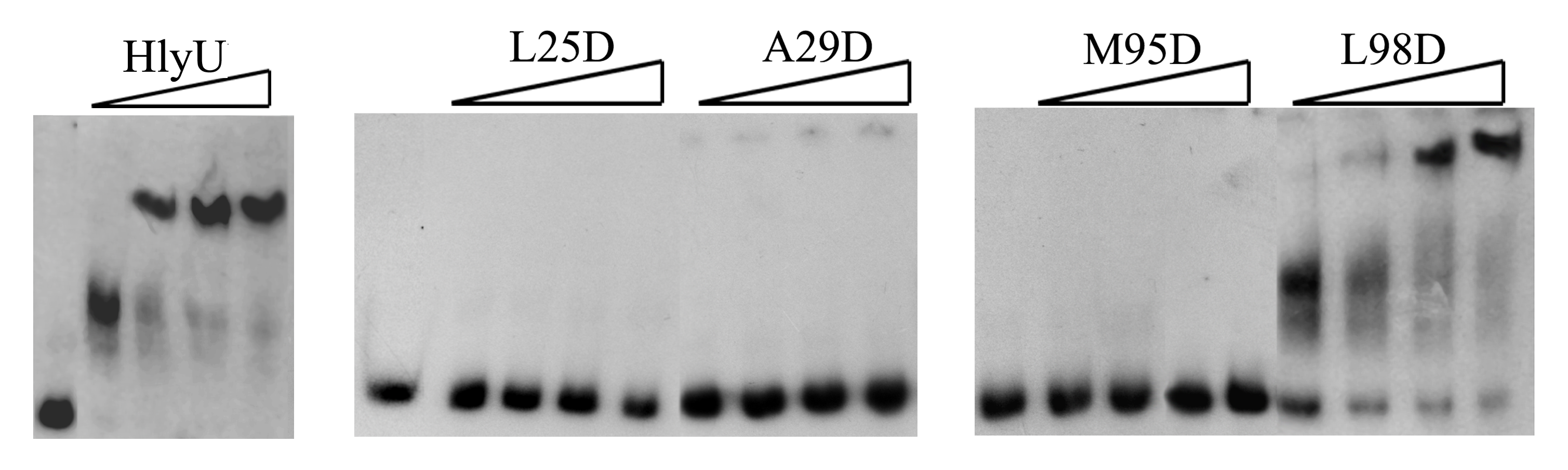
**

**(B)**

**
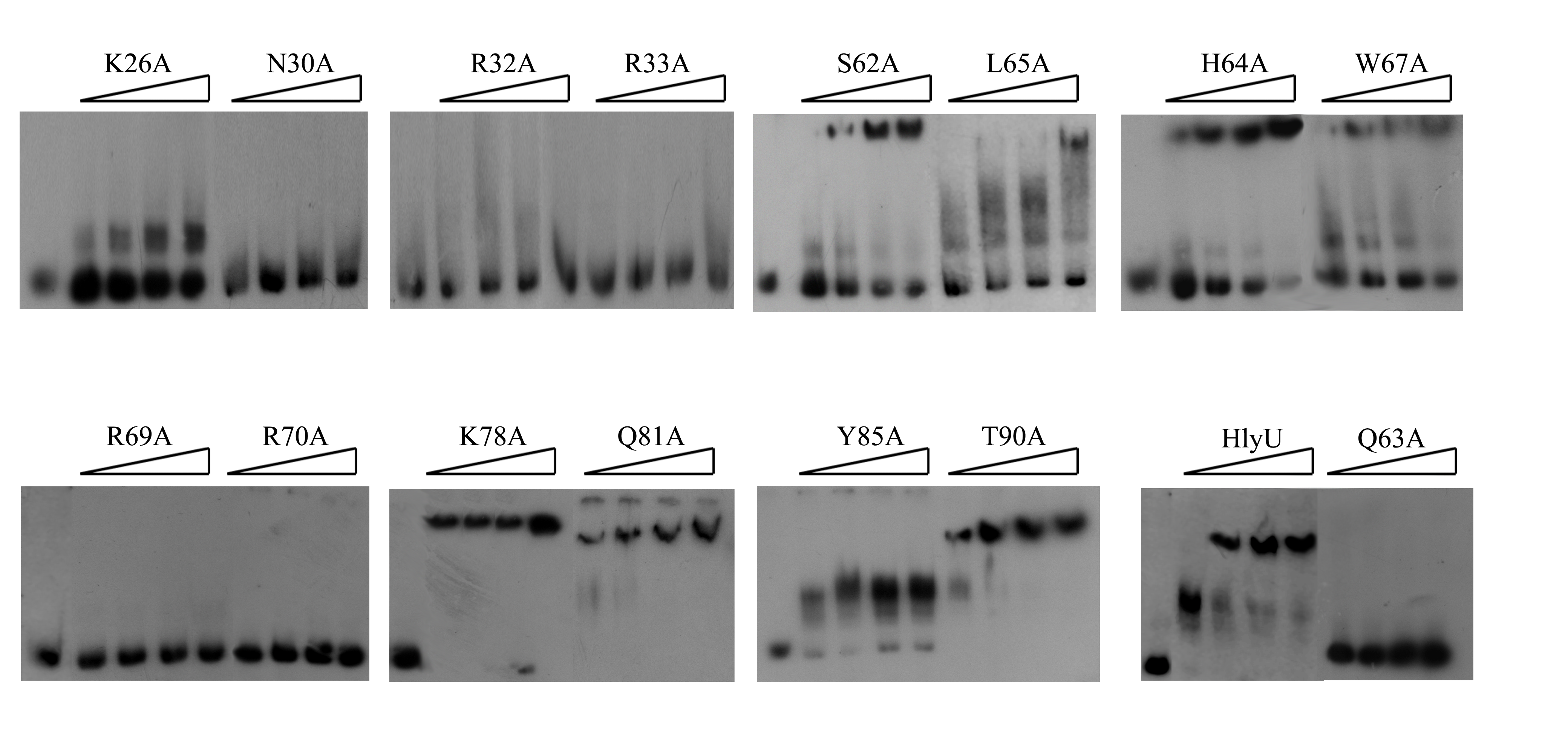
**

**Figure S4.** EMSA for binding of wild type HlyU_Vc compared to (A) self-contact mutants and (B) DNA binding mutants to the *hlyA* regulatory region. The leftmost lane for each gel contains the free probe (DNA6, Table 1). All the proteins have been used in the concentration range of 2 to 5 μM, with 1 μM increment in each step. These are extensions of Figures 3 and 4A which were carried out with lower protein concentrations.

(A)

(B)

(C)


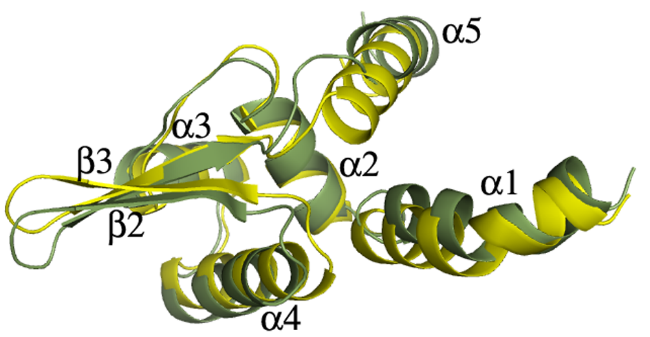


Figure S5. Analyses (using HELANAL-PLUS) of the structures obtained after MD simulation for HlyU_Vc (A) without DNA and (B) with DNA; (C) changes in structure after simulation of HlyU_Vc without DNA (yellow) and with DNA (green).


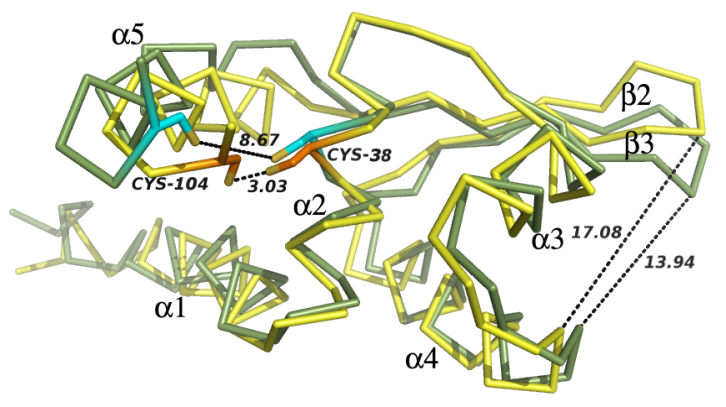


Figure S6. Correlation of the distance between the wing and the recognition helix and between Cys residues in HlyU_Vc without DNA (yellow ribbon with Cys in orange backbone) and with DNA (green ribbon with Cys in cyan backbone).

**
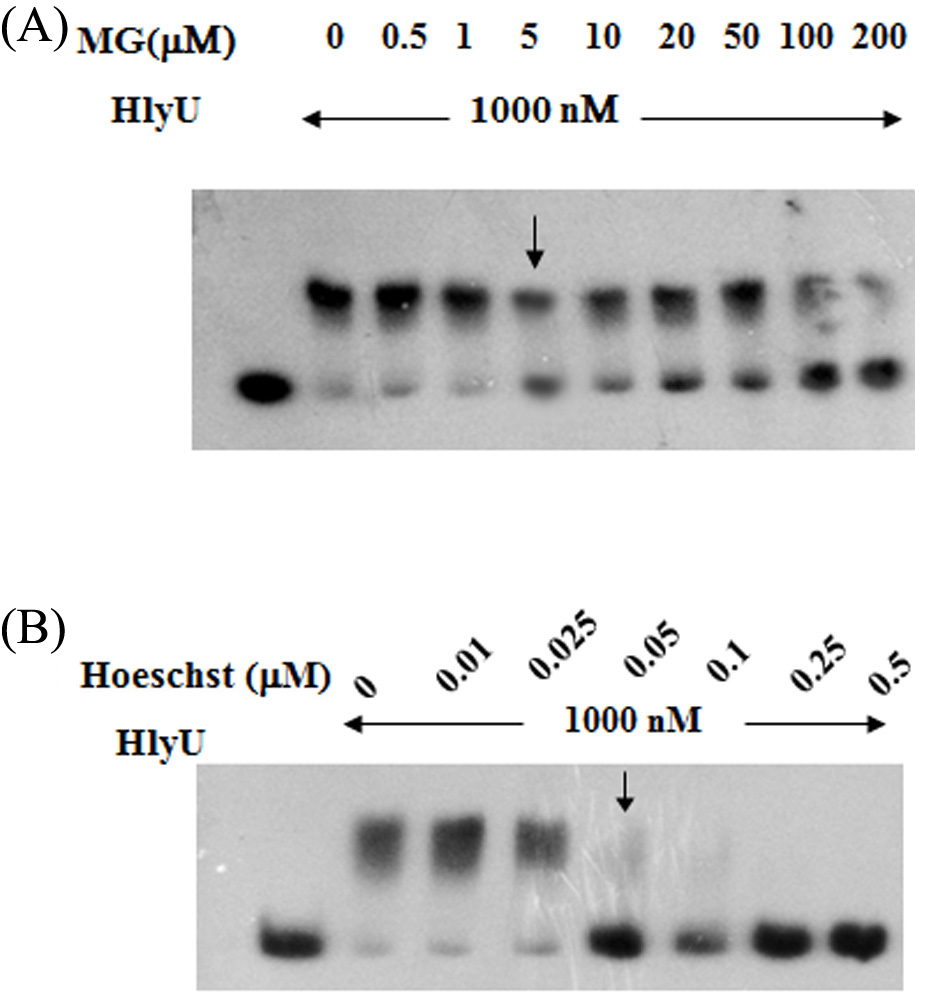
**

Figure S7. EMSA showing competition of the DNA-HlyU_Vc complex by (A) the major-groove-binding dye, methyl green (MG) and (B) the minor-groove-binding dye, Hoechst 33258. The vertical arrows indicate the start point of displacement of HlyU_Vc from DNA by the two dyes; the leftmost lane contains the free probe (DNA6, Table 1).

(A)


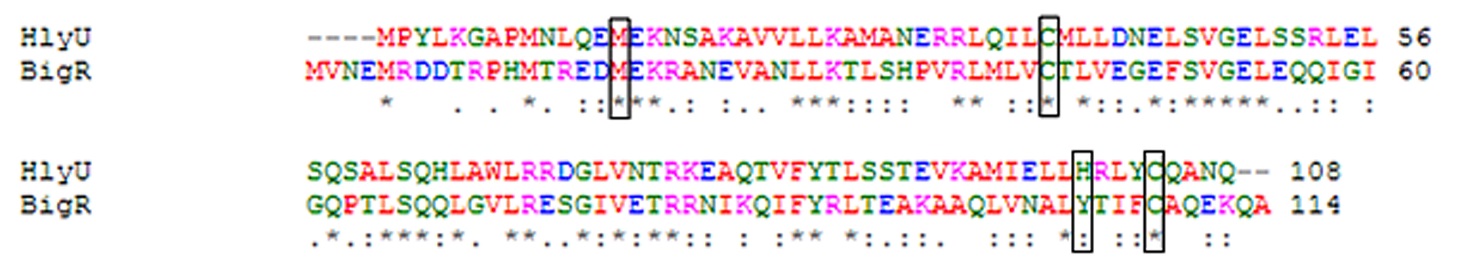


(B)


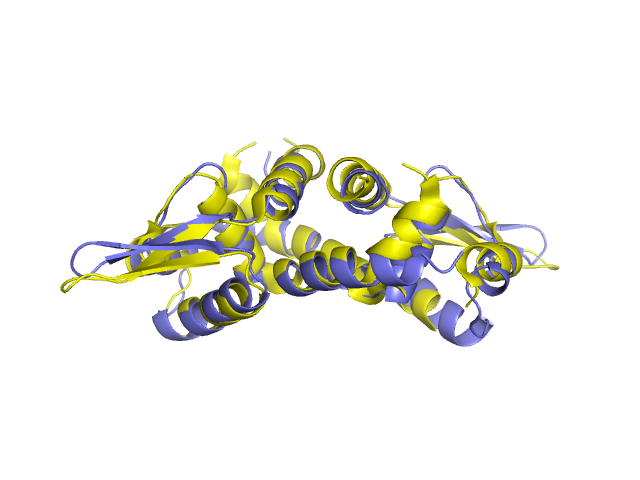


(C) (D)


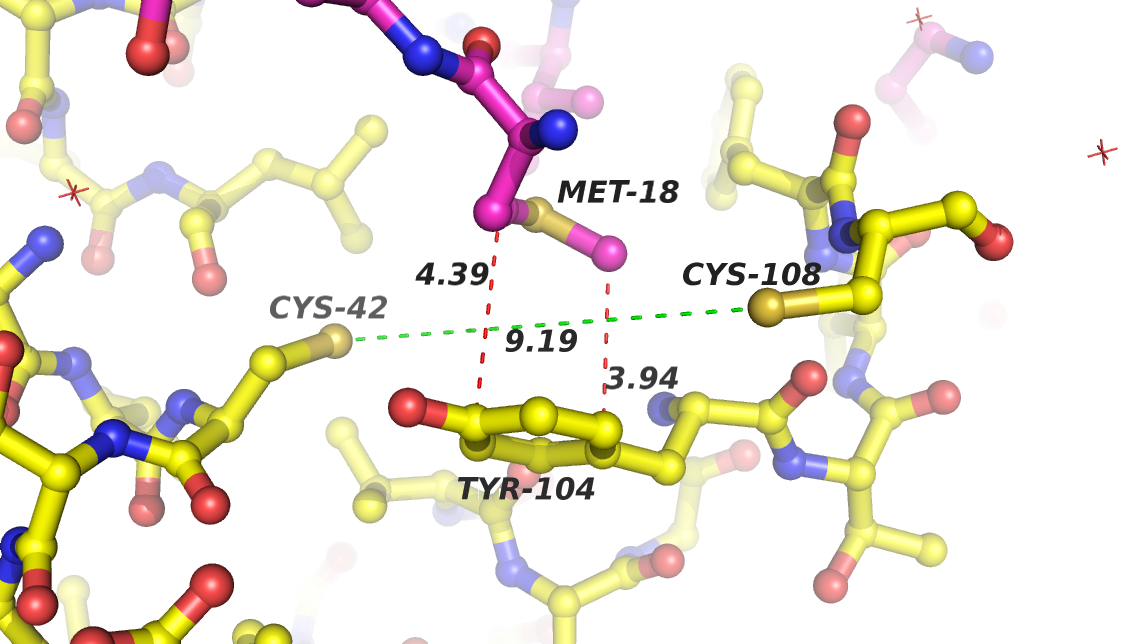

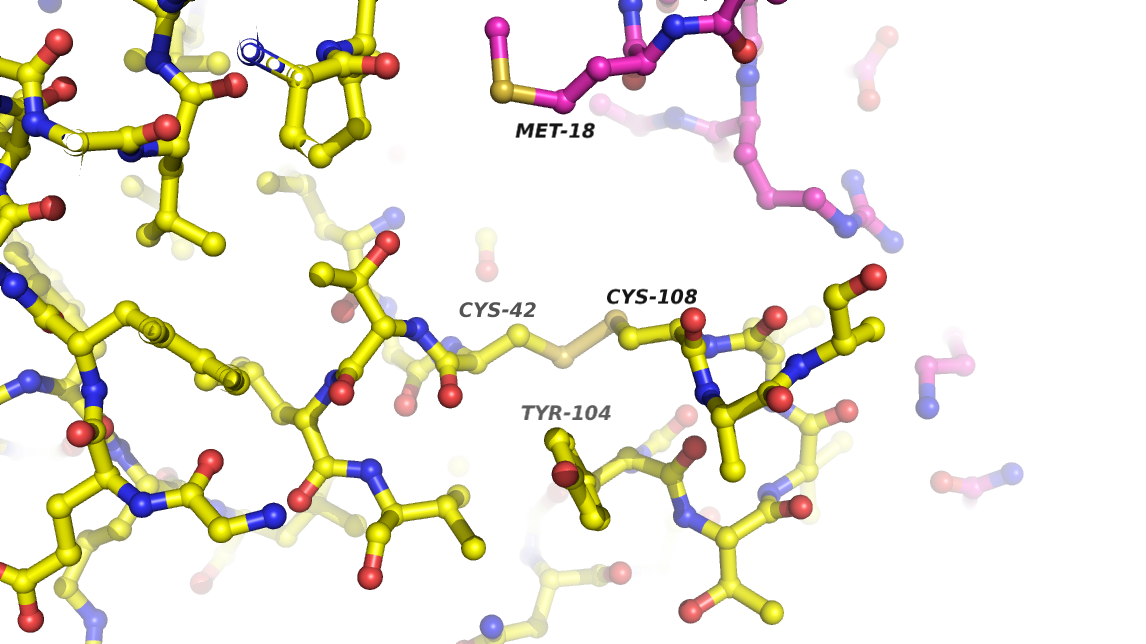


Figure S8. (A) Sequence alignment of BigR and HlyU_Vc showing the conserved positions of Cys, Met and the C-terminal aromatic residue (His or Tyr); (B) Structurally superposed crystal structures of HlyU_Vc (blue) and BigR (yellow); Cys-Cys and Met-His interactions in (C) the reduced and (D) the oxidized forms of BigR.


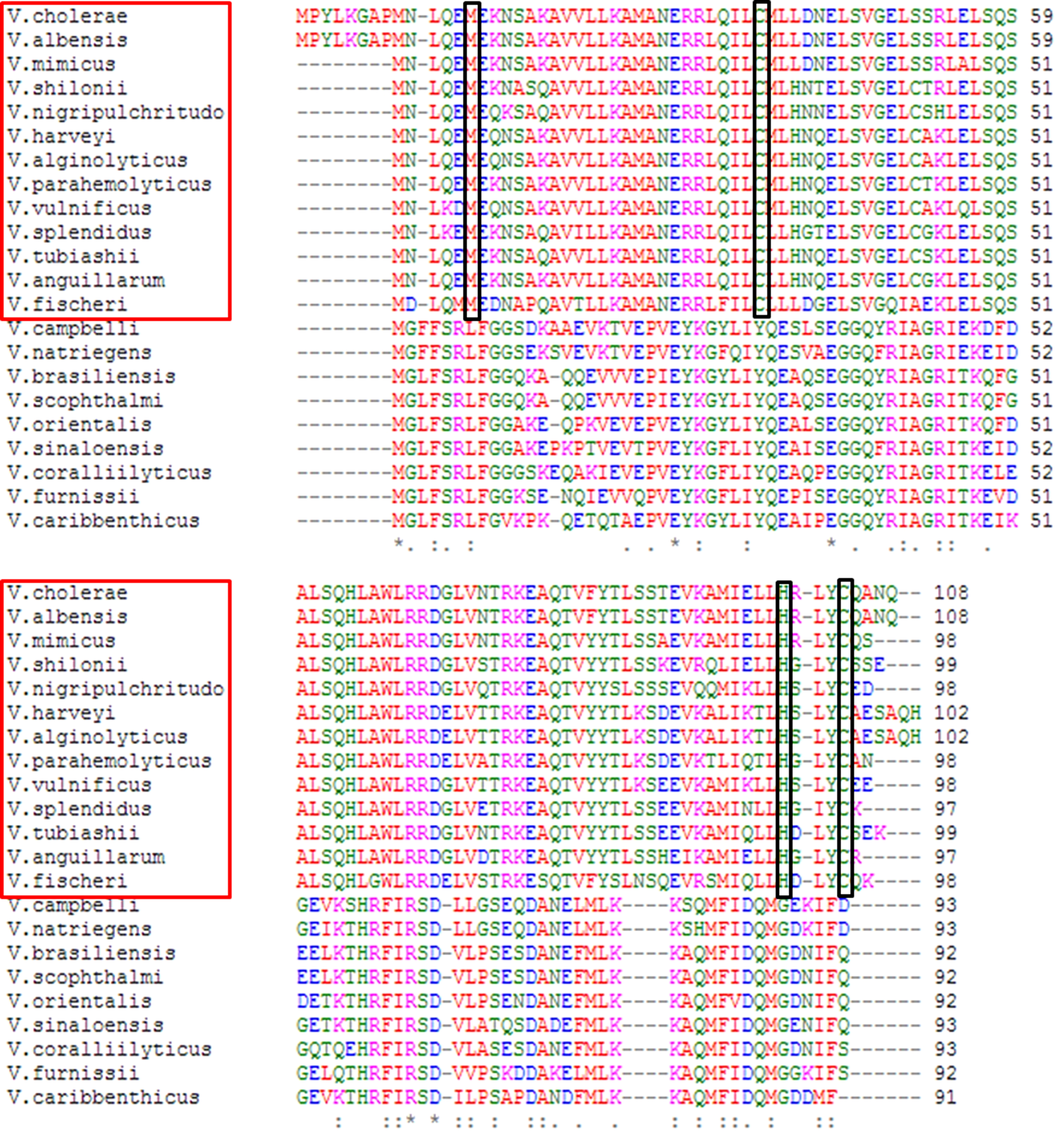


Figure S9. Multiple sequence alignment of HlyU from different *Vibrio* species. Black boxes indicate the conservation of the residues proposed to be involved in probable redox switch of HlyU among the species shown in red box.

Video SM1. Dynamics of HlyU_Vc in DNA bound state. Color codes: HlyU_Vc chains (A=green and B=blue), DNA strands (5′→3′ = red and 3′→5′ = gold).

Video SM2. Dynamics of HlyU_Vc in DNA bound state zooming into the environment of Cys residues. Protein backbone in green cartoon, residues are shown in stick (Cys = yellow, Met14 and His100 = standard element colors).

Video SM3. Dynamics of uncomplexed HlyU_Vc zooming into the environment of Cys residues. Protein backbone in green cartoon, residues are shown in stick (Cys = yellow, Met14 and His100 = standard element colors).

**References**

1. Saha, R.P. and Chakrabarti, P. (2006) Molecular modeling and characterization of Vibrio cholerae transcription regulator HlyU. *BMC structural biology*, **6**, 24.

2. Kim, S.K. and Norden, B. (1993) Methyl green. A DNA major-groove binding drug. *FEBS letters*, **315**, 61-64.

3. Portugal, J. and Waring, M.J. (1988) Assignment of DNA binding sites for 4',6-diamidine-2-phenylindole and bisbenzimide (Hoechst 33258). A comparative footprinting study. *Biochimica et biophysica acta*, **949**, 158-168.

4. Nielsen, P.E. (1991) Sequence-selective DNA recognition by synthetic ligands. *Bioconjugate chemistry*, **2**, 1-12.

5. Bontemps, J., Houssier, C. and Fredericq, E. (1975) Physico-chemical study of the complexes of "33258 Hoechst" with DNA and nucleohistone. *Nucleic acids research*, **2**, 971-984.
